# From Diaminosilylenes to Silapyramidanes: Making Sense of the Stability of Divalent Silicon Compounds

**DOI:** 10.1021/acsorginorgau.3c00041

**Published:** 2023-11-07

**Authors:** Kristian Torstensen, Abhik Ghosh

**Affiliations:** Department of Chemistry, UiT − The Arctic University of Norway, N-9037 Tromso̷, Norway

**Keywords:** silylene, carbene, diaminosilylene, decamethylsilicocene, silapyramidane

## Abstract

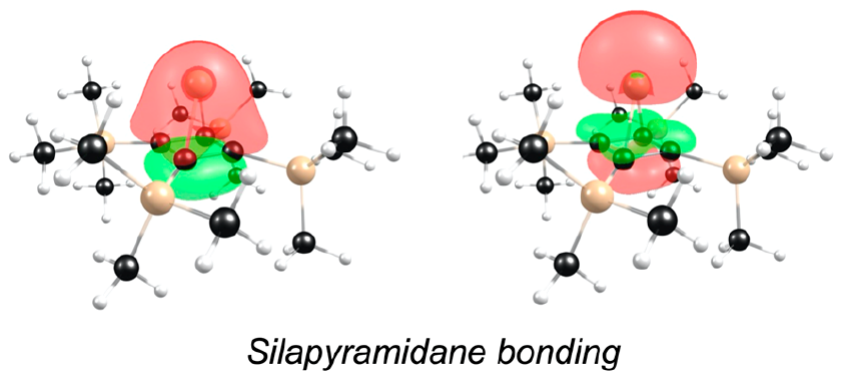

Since the discovery of decamethylsilicocene over three decades ago, chemists have successfully isolated a variety of divalent silicon compounds by orchestrating steric and electronic effects to their advantage. Two broad strategies of electronic stabilization appear to have been widely deployed, namely, π-conjugation as in diaminosilylenes and π-complexation as in decamethylsilicocene and silapyramidanes. Herein, we attempted to identify quantitative metrics for the electronic stabilization of silylenes. Singlet–triplet gaps and electron affinities, both physical observables, proved useful in this regard. Thus, the most stable silylenes exhibit unusually large singlet–triplet gaps and very low or negative gas-phase electron affinities. Both metrics signify low electrophilicity, i.e., a low susceptibility to nucleophilic attack. The chemical significance of the ionization potential associated with the Si-based lone pair, on the other hand, remains unclear.

Much of the modern renaissance in main-group chemistry has been driven by a desire to better understand and manipulate low-valent, low-oxidation-state and low-coordinate states of the elements.^1−10^ For silicon, a major landmark was the 1986 synthesis and structural chratacterization of decamethylsilicocene, Cp*_2_Si, the silicon analogue of a metallocene.^11−13^ Stable diaminosilylenes followed in a few years.^14−17^ Key developments in the twenty-first century include the synthesis and full characterization of the half-sandwich Cp*Si^+^ cation^18,19^ and, very recently, of the silapyramidane (Me_3_Si)_4_C_4_Si,^20,21^ consisting of a bare silicon atom atop a tetrakis(trimethylsilyl)cyclobutadiene base. The cyclobutadiene in the latter compound may be thought of as an aromatic dianion interacting with a Si^2+^ cation. Although each of these intriguing molecules has been described in molecular orbital terms, typically based on density functional theory (DFT), a comparative account of *quantitative* molecular properties appears to be lacking. Here we present a DFT (B3LYP^22,23^-D3^24^/def2QZVP)^25^ study of both simple silylenes and of the more unusual divalent silicon species mentioned above (Scheme 1). Besides optimized geometries, we calculated vertical and adiabatic ionization potentials (IPs), electron affinities (EAs) and singlet–triplet gaps (*E*_S-T_’s) in the hope that they would shed light on the special stability of π-complexes of decamethylsilicocene (Cp*_2_Si) and silapyramidanes (Table 1). Based on long-standing^26−30^ and recent^31−34^ calibration studies in our laboratory, the present level of theory was expected to yield vertical ionization potentials to within 0.1–0.2 eV of experimental values derived from photoelectron spectroscopy. Indeed, for parent silylene, SiH_2_, the calculated adiabatic IP and EA (Table 1) agree to within 0.1 eV with experimental values, 8.92 eV^35,36^ and 1.12 eV,^37^ respectively, providing excellent calibration for the present calculations.^38^ As described below, the calculated metrics (Table 1) provide valuable insight into, and indeed help quantify, the stability of key classes of divalent silicon species.

**Scheme 1:**
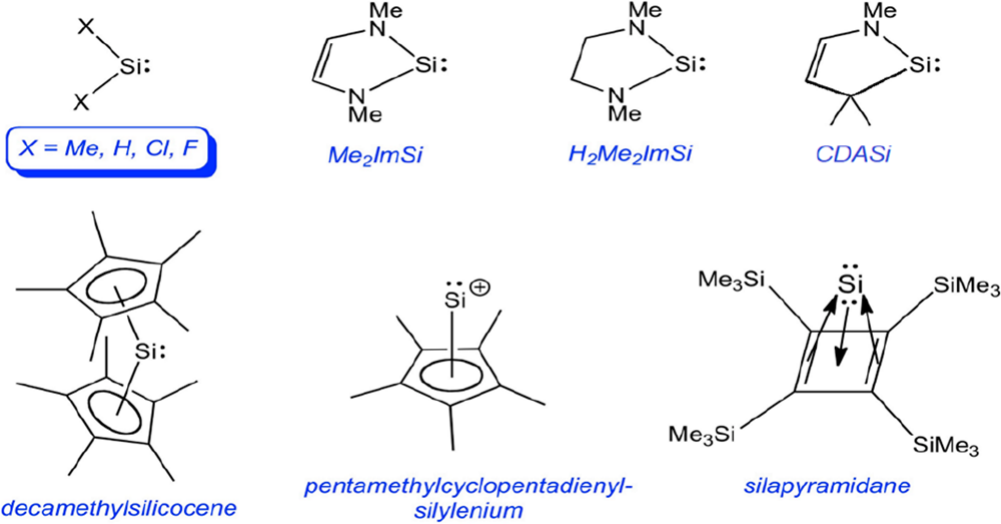
Molecules Studied in This Work

**Table 1:** Selected B3LYP molecular properties (eV).^25^

	IP		EA		*E*_S-T_	
molecule (point group)	vertical	adiabatic	vertical	adiabatic	vertical	adiabatic
SiH_2_ (*C*_2v_)	9.46	9.03	1.07	1.08	1.27	0.89
SiF_2_ (*C*_2v_)	11.13	10.73	0.12	0.28	3.29	3.18
SiCl_2_ (*C*_2v_)	10.01	9.55	1.00	1.25	2.46	2.29
SiMe_2_ (*C*_2_)	8.17	7.72	0.29	0.42	1.46	1.16
Me_2_ImSi (*C*_2v_)^a^	7.29 (8.64)	7.02 (8.32)	–0.89	–0.77	3.17	2.52
H_2_Me_2_ImSi (*C*_2_)^a^	7.53 (8.43)	7.39 (7.98)	–0.81	–0.55	3.36	3.12
CDASi (*C*_s_)^a^	7.77 (7.83)	7.45 (7.57)	–0.03	0.27	2.44	2.21
Cp*_2_Si (*C*_2_)	6.61	6.44	–0.81	0.00	3.59	2.02
silapyramidane (*C*_4_)	7.38	6.94	–0.93	–0.53	3.90	2.45
Cp*Si^+^ (*C*_5v_)					4.95	3.15

a For these systems, the first IP corresponds to ionization of the π-system. The second IP (in parentheses) involves ionization of the carbene lone pair.

The most salient difference between carbenes and silylenes is their singlet–triplet gap, which is much larger for silylenes. The difference reflects the much larger valence s-p energy difference for third period elements relative to second-period elements. Unlike simple carbenes such as methylene (CH_2_), which exhibit triplet ground states, virtually all silylenes are ground-state singlets^39−41^ (except for rare reports of triplet silylenes^42−46^). The vertical and adiabatic IPs of simple silylenes such as SiF_2_ and SiCl_2_ are similar to those calculated for the analogous, nucleophilic carbenes (Table 1).^15,31^ As in the case of carbenes,^31^ substituents have a strong influence in determining the IP associated with the Si-based lone pairs, which increases by >4 eV on going from Cp*_2_Si to SiF_2_. In yet another similarity with analogous carbenes,^15,31^ the lowest IP of the diaminosilylenes Me_2_ImSi and H_2_Me_2_ImSi and of the cyclic dialkyl-amino-silylene CDASi (Scheme 1 and Table 1) was found to correspond to ionization of the π-system; the second IP of these systems corresponds to ionization of the silylene lone pairs. Overall, however, the IPs of the silylenes studied do not exhibit any obvious correlation with known chemical behavior or reactivity. On the other hand, the simpler silylenes exhibit positive electron affinities, suggesting susceptibility to nucleophilic attack.

The present calculations underscore two unusual features of divalent Si π-complexes. First, they exhibit unusually large singlet–triplet gaps relative to simple silylenes. For Cp*_2_Si, the silapyramidane, and the Cp*Si^+^ cation, the vertical singlet triplet gap ranges from 3.6 eV to an astonishing 4.95 eV. Admittedly, the adiabatic gaps are much lower, reflecting large geometry changes in the triplet state, but they are still large, >2 eV. Second, aside from the Cp*Si^+^ cation, the π-complexes decamethylsilicocene and the silapyramidane do not exhibit a positive vertical electron affinity—in fact, the vertical EAs are strongly negative! This property is also shared by Me_2_ImSi and its saturated dihydro counterpart H_2_Me_2_ImSi. In other words, these molecules are not expected to exhibit electron attachment in the gas phase and, by extension, may also be expected to withstand nucleophilic attack, at least to a degree, in solution.

The orbital interactions responsible for the large singlet–triplet gaps and low (or negative) electron affinities of stable silylenes are fairly well-understood.^13,15^ In the case of diaminosilylenes, for example, these are allyl-type π-conjugative interactions, with the LUMO identified as the fully antibonding p-orbital of the N–Si–N unit. In the case of π-complexes such as decamethylsilicocene and the silapyramidane, strong bonding interactions involving Si greatly increase the HOMO–LUMO gap of the carbocycle ligands. In the case of the silapyramidane,^20^ for example, the fully bonding π-orbital of the cyclobutadiene moiety interacts head-on with a Si 3s/3p_z_ hybrid orbital (Figure 1). The resulting bonding and antibonding MOs constitute the HOMO-42 and HOMO-2 of the silapyramidane (both of which transform as ‘a’ under *C*_4_). The stability of the HOMO-2 reflects its predominant Si 3s character. The two degenerate π-MOs of the cyclobutadiene are also suitably aligned to bond with the Si 3p_x_ and 3p_y_ orbitals to generate a pair of ‘e’ MOs, the HOMOs of the silapyramidane. In contrast, the fully antibonding π-orbital of the cyclobutadiene cannot (on account of its symmetry) engage in any energy-lowering interaction with Si orbitals and may be identified as the molecular LUMO (Figure 1).

**Figure 1:**
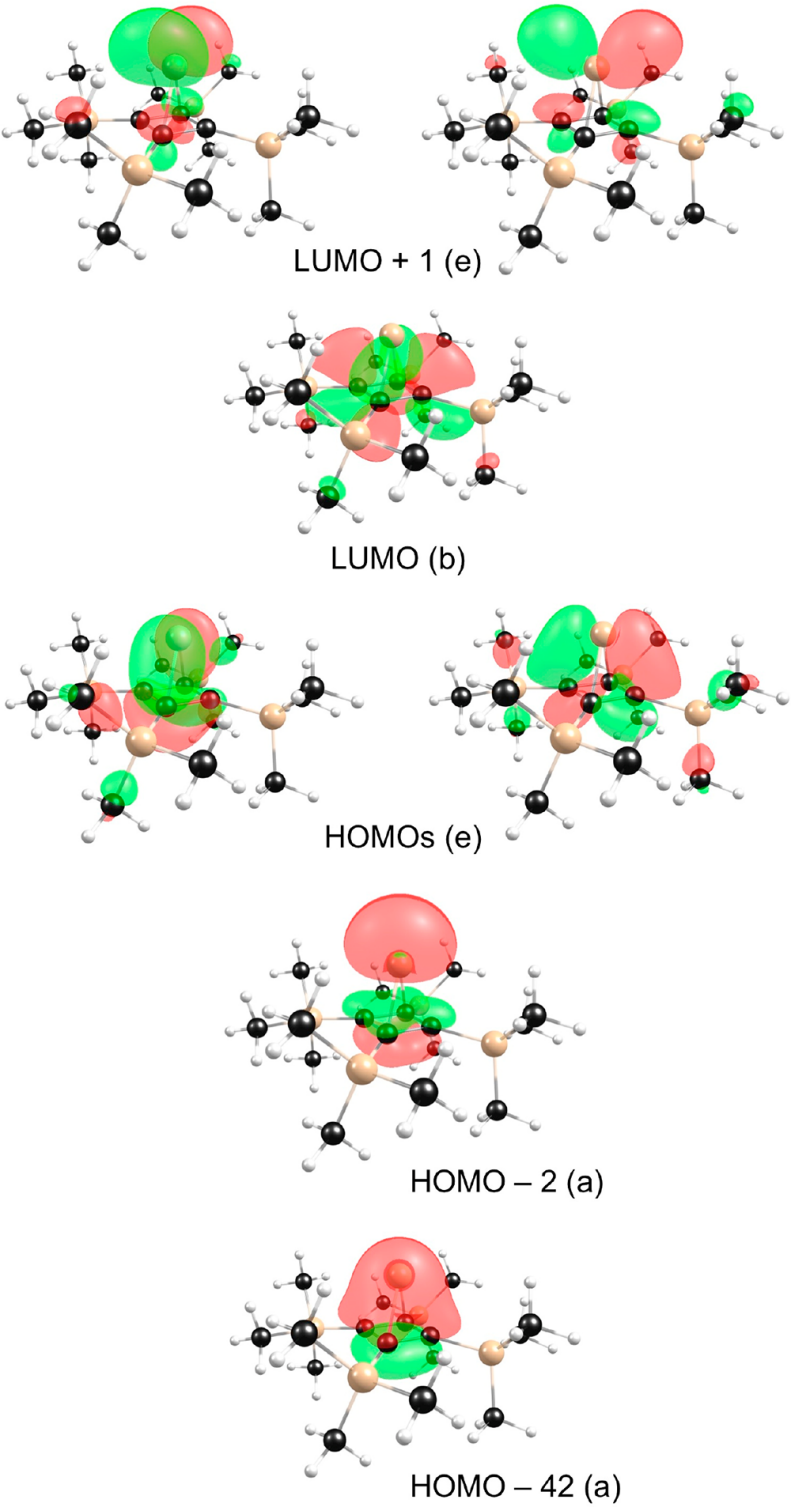
Selected Kohn–Sham MOs depicting Si-cyclobutadiene bonding in the silapyramidane studied.

In conclusion, at least two quantitative metrics, the singlet–triplet gap and the electron affinity, appear to correlate with experimentalists’ qualitative ideas about the stability of silylenes. While a high singlet–triplet gap is a well-known measure of molecular stability, a low or negative electron affinity is indicative of low electrophilicity or low susceptibility to nucleophilic attack. Both π conjugation (especially with amino substituents) and π complexation emerge as comparably effective strategies that afford stable divalent silicon compounds.

## Data Availability

The data underlying this study are available in the published article and its Supporting Information.

## Supporting Information Available

Additional computational results; optimized Cartesian coordinates (PDF)
