# Supplementary material for: From Diaminosilylenes to Silapyramidanes: Making Sense of the Stability of Divalent Silicon Compounds
Source: ACS Org Inorg Au. 2023 Nov 7;4(1):102–5. doi: 10.1021/acsorginorgau.3c00041 (PMC10853992; doi:10.1021/acsorginorgau.3c00041)
Supplement: Supplementary file 1 — gg3c00041_si_001.pdf [file gg3c00041_si_001.pdf]

Supporting Information

From Diaminosilylenes to Silapyramidanes: Making Sense of the Stability of Divalent Silicon Compounds

Kristian Torstensen and Abhik Ghosh*

Department of Chemistry, UiT – The Arctic University of Norway, N-9037 Tromsø, Norway

Contents **Page**

A. Highlights of B3LYP-D3 /def2QZVP optimized geometries and frontier Mos S2

B. W1RO energetics S3

C. DFT optimized Cartesian coordinates S4

A. Highlights of B3LYP-D3 /def2QZVP optimized geometries and frontier MOs.

Molecule & Point group		Geometry (Å, °)	HOMO	LUMO
SiH_2	C_{2v}	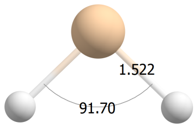	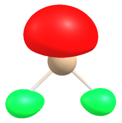	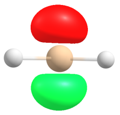
SiF_2	C_{2v}	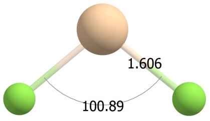	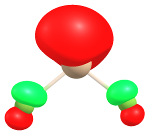	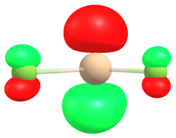
$SiCl_2$	C_{2v}	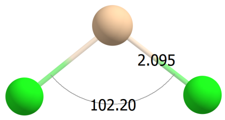	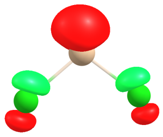	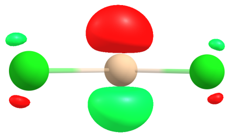
$SiMe_2$	C_s	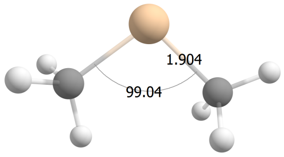	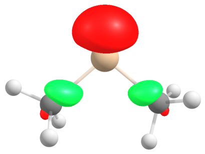	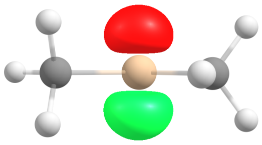
Me_2ImSi	C_{2v}	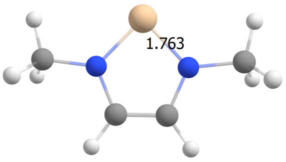	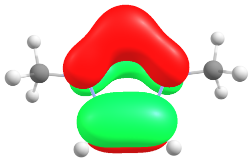	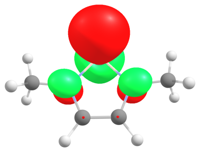
H_2Me_2ImSi	C_2	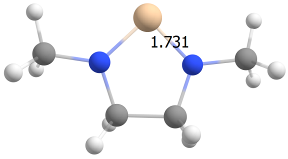	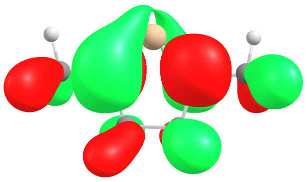	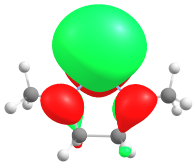
$Cp*_2Si$	C_2	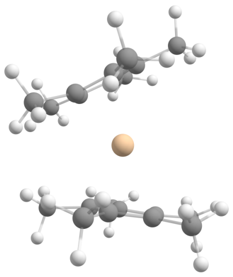	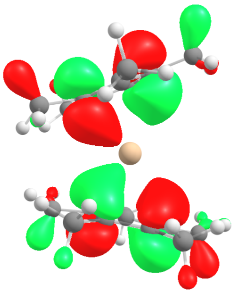	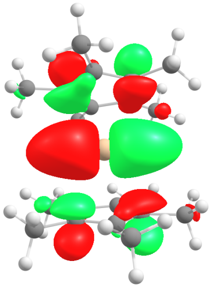

Silapyramidane	C_4	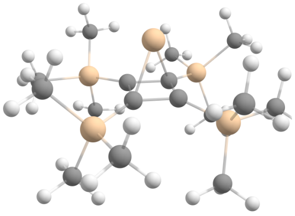	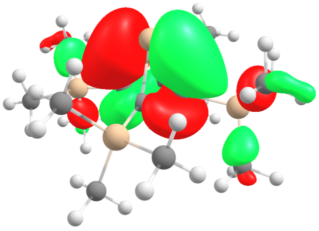	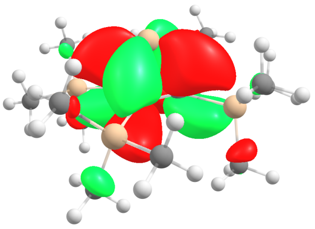
Cp^*Si^+	C_{5v}	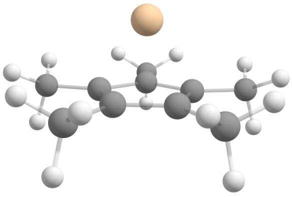	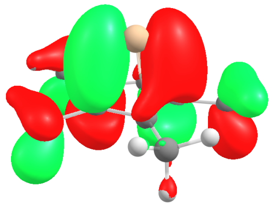	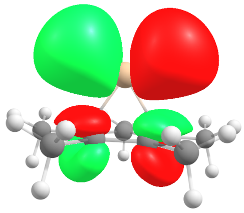

B. W1RO energetics

Table S1. W1RO energetics (eV).

Molecule (Point group)	IP Adiabatic	EA Adiabatic	$E_{S\text{-}T}$ Adiabatic
SiH_2 (C_{2v})	9.12	1.05	0.92
SiF_2 (C_{2v})	10.80	0.22	3.26
$SiCl_2$ (C_{2v})	9.67	1.17	2.38
$SiMe_2$ (C_2)	7.86	0.42	1.20

C. B3LYP-D3/def2-QZVP optimized Cartesian coordinates (Å)

1. SiH_2

H	-0.495095000	0.746702000	0.849240000
H	-0.495101000	1.344075000	-1.251226000
Si	0.116889000	1.877862000	0.035760000

2. SiF_2

F	2.048480000	1.285649000	0.000000000
F	-0.427524000	1.220289000	0.000000000
Si	0.783484000	2.275552000	0.000000000

3. $SiCl_2$

Cl	-0.677650000	1.243107000	-1.839947000
Cl	-0.677641000	0.351039000	1.296720000
Si	0.081986000	1.830383000	0.022257000

4. $SiMe_2$

Si	0.000000000	0.000000000	0.754862000
C	0.000000000	1.436527000	-0.496817000
H	-0.653368000	1.260658000	-1.355494000
H	1.022139000	1.501239000	-0.894527000
H	-0.237201000	2.402451000	-0.053110000
C	0.000000000	-1.436527000	-0.496817000
H	-1.022139000	-1.501239000	-0.894527000
H	0.237201000	-2.402451000	-0.053110000
H	0.653368000	-1.260658000	-1.355494000

5. Me_2ImSi

C	0.000000000	-0.675986000	-1.334232000
C	0.000000000	0.675986000	-1.334232000
C	0.000000000	2.658842000	0.106109000
C	0.000000000	-2.658842000	0.106109000
H	-0.885432000	3.108692000	-0.347404000
H	0.885432000	3.108692000	-0.347404000
H	0.000000000	2.905428000	1.166128000
H	0.885432000	-3.108692000	-0.347404000
H	-0.885432000	-3.108692000	-0.347404000
H	0.000000000	-2.905428000	1.166128000
H	0.000000000	-1.310171000	-2.206681000
H	0.000000000	1.310171000	-2.206681000
N	0.000000000	1.214304000	-0.060373000
N	0.000000000	-1.214304000	-0.060373000
Si	0.000000000	0.000000000	1.218340000

6. H_2Me_2ImSi

C	-0.728047000	0.242711000	-1.372080000
C	0.728047000	-0.242711000	-1.372080000

C	-2.629334000	0.403231000	0.186126000
C	2.629334000	-0.403231000	0.186126000
H	-1.338247000	-0.348308000	-2.061206000
H	-0.792375000	1.287316000	-1.703765000
H	1.338247000	0.348308000	-2.061206000
H	0.792375000	-1.287316000	-1.703765000
H	-3.254474000	-0.313004000	-0.356998000
H	-2.895080000	1.406924000	-0.167251000
H	-2.887943000	0.340642000	1.242596000
H	3.254474000	0.313004000	-0.356998000
H	2.895080000	-1.406924000	-0.167251000
H	2.887943000	-0.340642000	1.242596000
N	-1.223379000	0.123363000	-0.001651000
N	1.223379000	-0.123363000	-0.001651000
Si	0.000000000	0.000000000	1.216505000

7. CDASi

C	1.374305000	0.437678000	0.000000000
C	1.308078000	-0.892943000	0.000000000
N	0.011538000	-1.452797000	0.000000000
C	0.048630000	1.149788000	0.000000000
C	-0.101261000	-2.903641000	0.000000000
H	0.372251000	-3.335822000	0.884717000
H	0.372251000	-3.335822000	-0.884717000
H	-1.151003000	-3.189005000	0.000000000
H	2.314606000	0.974935000	0.000000000
H	2.154795000	-1.567566000	0.000000000
Si	-1.279171000	-0.289647000	0.000000000
C	-0.101261000	2.029825000	-1.258857000
H	0.002653000	1.451255000	-2.178309000
H	0.669217000	2.806250000	-1.272615000
H	-1.071196000	2.529857000	-1.283129000
C	-0.101261000	2.029825000	1.258857000
H	0.002653000	1.451255000	2.178309000
H	-1.071196000	2.529857000	1.283129000
H	0.669217000	2.806250000	1.272615000

8. Decamethylsilicocene

C	0.000000000	2.524704000	0.063257000
C	0.948470000	2.372481000	1.100502000
C	2.030054000	1.610273000	0.605768000
C	1.763456000	1.284629000	-0.751120000
C	0.496597000	1.854352000	-1.093936000
C	2.713448000	0.607576000	-1.692394000
C	3.255457000	1.218265000	1.372579000
C	0.816005000	2.902621000	2.495231000
C	-1.278226000	3.302187000	0.138073000
C	1.278226000	-3.302187000	0.138073000

C	0.000000000	-2.524704000	0.063257000
C	-0.816005000	-2.902621000	2.495231000
C	-0.948470000	-2.372481000	1.100502000
C	-0.496597000	-1.854352000	-1.093936000
C	-2.030054000	-1.610273000	0.605768000
C	-1.763456000	-1.284629000	-0.751120000
C	-3.255457000	-1.218265000	1.372579000
C	-2.713448000	-0.607576000	-1.692394000
C	-0.084138000	1.954285000	-2.472478000
C	0.084138000	-1.954285000	-2.472478000
H	3.233840000	-0.224764000	-1.218096000
H	3.478209000	1.304792000	-2.045020000
H	2.202662000	0.217098000	-2.569622000
H	3.721029000	0.325164000	0.958252000
H	3.030774000	1.015786000	2.419618000
H	4.007311000	2.012116000	1.353517000
H	-0.226995000	2.977085000	2.801010000
H	1.250161000	3.902019000	2.587012000
H	1.324189000	2.265469000	3.218381000
H	-1.710580000	3.274856000	1.137692000
H	-2.026302000	2.916551000	-0.553443000
H	-1.118129000	4.354067000	-0.114684000
H	2.026302000	-2.916551000	-0.553443000
H	1.118129000	-4.354067000	-0.114684000
H	1.710580000	-3.274856000	1.137692000
H	0.226995000	-2.977085000	2.801010000
H	-1.250161000	-3.902019000	2.587012000
H	-1.324189000	-2.265469000	3.218381000
H	-4.007311000	-2.012116000	1.353517000
H	-3.721029000	-0.325164000	0.958252000
H	-3.030774000	-1.015786000	2.419618000
H	-2.202662000	-0.217098000	-2.569622000
H	-3.233840000	0.224764000	-1.218096000
H	-3.478209000	-1.304792000	-2.045020000
H	0.255843000	2.867139000	-2.969356000
H	-1.172261000	1.984802000	-2.454828000
H	0.211662000	1.118181000	-3.102842000
H	-0.255843000	-2.867139000	-2.969356000
H	1.172261000	-1.984802000	-2.454828000
H	-0.211662000	-1.118181000	-3.102842000
Si	0.000000000	0.000000000	0.279931000

9. Pentamethylcuclopentadienylsilylenium

C	-1.070089000	-0.585302000	-0.281022000
C	0.225980000	-1.198583000	-0.281022000
C	1.209752000	-0.155463000	-0.281022000
C	0.521688000	1.102502000	-0.281022000
C	-0.887331000	0.836847000	-0.281022000

C	1.161012000	2.453597000	-0.331923000
C	-1.974737000	1.862391000	-0.331923000
C	-2.381466000	-1.302576000	-0.331923000
C	0.502910000	-2.667428000	-0.331923000
C	2.692282000	-0.345984000	-0.331923000
H	1.301843000	2.746004000	-1.373799000
H	2.137345000	2.460104000	0.145417000
H	0.544912000	3.213379000	0.141665000
H	-2.209313000	2.086688000	-1.373799000
H	-1.679223000	2.792950000	0.145417000
H	-2.887718000	1.511230000	0.141665000
H	-2.667273000	-1.456360000	-1.373799000
H	-3.175161000	-0.733967000	0.145417000
H	-2.329620000	-2.279387000	0.141665000
H	0.560848000	-2.986768000	-1.373799000
H	-0.283135000	-3.246566000	0.145417000
H	1.447934000	-2.919969000	0.141665000
H	3.013896000	-0.389564000	-1.373799000
H	3.000174000	-1.272522000	0.145417000
H	3.224492000	0.474747000	0.141665000
Si	0.000000000	0.000000000	1.498444000

10. Silapyramidane

C	-0.962877000	0.413751000	0.149841000
C	0.413751000	0.962877000	0.149841000
C	0.962877000	-0.413751000	0.149841000
C	-0.413751000	-0.962877000	0.149841000
C	2.528465000	3.010088000	1.180946000
C	0.000000000	4.083127000	0.022341000
C	1.920301000	2.676630000	-1.851902000
C	-3.010088000	2.528465000	1.180946000
C	-2.676630000	1.920301000	-1.851902000
C	-4.083127000	0.000000000	0.022341000
C	-2.528465000	-3.010088000	1.180946000
C	-1.920301000	-2.676630000	-1.851902000
C	0.000000000	-4.083127000	0.022341000
C	2.676630000	-1.920301000	-1.851902000
C	3.010088000	-2.528465000	1.180946000
C	4.083127000	0.000000000	0.022341000
H	3.321327000	2.270005000	1.221645000
H	2.987241000	3.978779000	0.971313000
H	2.072401000	3.067830000	2.170426000
H	-0.341839000	4.231272000	1.045865000
H	0.537058000	4.987701000	-0.272290000
H	-0.870888000	3.996768000	-0.622879000
H	1.115517000	2.612523000	-2.586322000
H	2.464181000	3.605151000	-2.034869000
H	2.599422000	1.846579000	-2.040609000

H	-3.067830000	2.072401000	2.170426000
H	-2.270005000	3.321327000	1.221645000
H	-3.978779000	2.987241000	0.971313000
H	-3.605151000	2.464181000	-2.034869000
H	-1.846579000	2.599422000	-2.040609000
H	-2.612523000	1.115517000	-2.586322000
H	-4.231272000	-0.341839000	1.045865000
H	-4.987701000	0.537058000	-0.272290000
H	-3.996768000	-0.870888000	-0.622879000
H	-2.072401000	-3.067830000	2.170426000
H	-3.321327000	-2.270005000	1.221645000
H	-2.987241000	-3.978779000	0.971313000
H	-2.599422000	-1.846579000	-2.040609000
H	-1.115517000	-2.612523000	-2.586322000
H	-2.464181000	-3.605151000	-2.034869000
H	0.870888000	-3.996768000	-0.622879000
H	0.341839000	-4.231272000	1.045865000
H	-0.537058000	-4.987701000	-0.272290000
H	2.612523000	-1.115517000	-2.586322000
H	3.605151000	-2.464181000	-2.034869000
H	1.846579000	-2.599422000	-2.040609000
H	3.978779000	-2.987241000	0.971313000
H	3.067830000	-2.072401000	2.170426000
H	2.270005000	-3.321327000	1.221645000
H	4.987701000	-0.537058000	-0.272290000
H	3.996768000	0.870888000	-0.622879000
H	4.231272000	0.341839000	1.045865000
Si	0.000000000	0.000000000	1.900101000
Si	1.208114000	2.647907000	-0.107856000
Si	2.647907000	-1.208114000	-0.107856000
Si	-1.208114000	-2.647907000	-0.107856000
Si	-2.647907000	1.208114000	-0.107856000
